# Assessing sexuality of patients on dialysis and renal transplant: A Tunisian study

**DOI:** 10.1192/j.eurpsy.2023.2325

**Published:** 2023-07-19

**Authors:** G. Tlili, A. Loghmari, K. Bouassida, M. Ben Othmen, E. Acacha, W. Ben Abdallah, M. Kahloul, W. Hmida, M. Jaidane

**Affiliations:** 1 Urology; 2 Anesthesiology department, Sahloul teachin Hospital Sousse, Sousse, Tunisia

## Abstract

**Introduction:**

Chronic renal failure is a public health issue. It leads to a degradation of physical integrity, hormonal disorders and a great psychological impact, which can lead to sexual disorders.

**Objectives:**

The aim of this study was to identify risk factors of sexual dysfunction in patients on dialysis and renal transplant patients.

**Methods:**

This is a cross-sectional survey conducted in nephrology department of Sahloul teaching hospital (Sousse) and Fattouma Bourguiba teaching hospital (Monastir) over two-month period. Patients on dialysis and renal transplant patients were included. Sexuality was assessed by FSFI and IIEF 15

**Results:**

This study enrolled 137 patients (99 patients with chronic renal failure and 38 renal transplant patients). The incidence of erectile dysfunction of men on dialysis was 57% and was associated to a decrease in testosterone level (11%) and an increase in LH level (50%). Its main risk factors were age (p=0.000), the duration (p=0.009), the cardiovascular diseases (p=0.03), the anxiety (p=0.000), the depression (p=0.000) and the different aspects of erectile dysfunction (p=0.000. In women on dialysis, the most common sexual disorder noticed was sexual arousal disorder (78.8%).

In the transplant, erectile dysfunction was found in 18.2% of transplant men and was associated to age was a predictive factor (p=0.03). In women, orgasm and desire disorders were the most common (69%).

Renal transplantation improved erectile dysfunction in men with IIEF score rising from 14 to 27 (p=0.021). It also improved sexual life in women: increase of desire (p=0.042) and orgasm scores (p=0.034).

**Conclusions:**

Sexual disorders remain common in patients on dialysis and with renal transplant. Their management requires a systematic screening to improve patients’ outcome

**Disclosure of Interest:**

None Declared

